# Understanding the *WHO health workforce support and safeguards list* *2023*

**DOI:** 10.2471/BLT.23.290191

**Published:** 2023-06-01

**Authors:** Giorgio Cometto, Mathieu Boniol, Agya Mahat, Khassoum Diallo, James Campbell

**Affiliations:** a Health Workforce Department, World Health Organization, avenue Appia 20, 1211 Geneva 27, Switzerland.

Health workforce challenges hinder progress towards universal health coverage, improved health outcomes^1^ and health security. The global health workforce shortage is declining,^2^ but progress is slower in the African and Eastern Mediterranean regions and Small Island Developing States. International migration of health workers, when not adequately managed, can exacerbate pre-existing inequalities, further depleting the availability of health workers in countries already affected by shortages.

To mitigate these challenges, the World Health Assembly adopted in 2010 the WHO Global Code of Practice on the International Recruitment of Health Personnel. The code has two major objectives: first, to guide international cooperation in the ethical management of health worker migration; second, to catalyse action and investment in the health systems of developing countries facing health workforce shortages.^3^ One of the core provisions of the code is to discourage active international recruitment from low- and middle-income countries affected by workforce challenges.

In 2020, an independent review of the relevance and effectiveness of the code documented its continued relevance, as international migration of health personnel has continued to rise.^4^ As to effectiveness, examples of successful implementation exist, but also areas where the code’s impact could be increased. For example, one of the review’s recommendations was to regularly update the list of countries facing severe health workforce challenges.

In response, the World Health Organization (WHO) produced a *Health workforce support and safeguards list*, identifying countries with low health workforce density and a low coverage of essential health services.^5^ The list is dynamic, with anticipated updates every three years reflecting country progress on health workforce density and service coverage. The 2023 update^6^ recognized the increased vulnerabilities caused by the coronavirus disease 2019 (COVID-19) pandemic, which posed additional stressors on health systems and the health workforce, and contributed to the acceleration of international migration of health personnel.^7^

The 55 countries identified in the 2023 revision should be prioritized for health workforce support by governments and the international community, and safeguarded by discouraging active international recruitment. In addition, government-to-government agreements should be informed by a health labour market analysis; adopt provisions promoting adequate domestic health workforce supply; engage health sector stakeholders; and specify proportional benefits to health systems of source and destination countries.^6^

Some people lament that the code – or the list – would limit free movement of health personnel, impeding the pursuit of employment and career development opportunities abroad, in the face of limited job opportunities in countries of origin.^8^ This interpretation is incorrect and such misperceptions should be dispelled: neither the code nor the list place any restriction on voluntary health worker mobility.

Free movement of individuals across countries is recognized by other international instruments,^9^ and continues to take place according to the relevant legislation and policies of source and destination countries. The code and the list have no provisions limiting the individual pursuit of employment opportunities in other countries. Rather, they aim to discourage systematic and proactive approaches by employers or recruiting agencies to recruit large numbers of health workers from countries of origin with workforce vulnerabilities to address shortages in destination countries.

In an attempt to reconcile the right of health workers to migrate with the local population’s right to health, the code and associated list represent a framework for ethical management of international recruitment of health personnel, with investments in, and benefits for, health systems of countries of origin, upholding the labour rights of migrant health workers, and encouraging destination countries to overcome the dependency on international recruitment. Encouragingly, some high-income destination countries have adopted national codes of practice^10^ directly inspired by the code.

International migration of health personnel and the challenges and opportunities it poses have to be interpreted and addressed through the lens of the health labour market. Migration results from large differentials in working conditions and wages across countries; it is a long-term phenomenon driven by fundamental market forces, which may accelerate further due to population and health workforce ageing trends in some regions.^11^

Therefore, migration should be managed through a combination of complementary policy responses. Destination countries should increase production of health workers to meet domestic needs; source countries should invest through domestic financing and development assistance, and adopt workforce policies to absorb the health workers in their health systems and to improve working conditions, including fair remuneration, to enhance retention. Planning and education policies in source countries should take into account the attrition in health personnel workforce due to international migration. Finally, development partners and international organizations should prioritize technical and financial support to countries in the list, and other low- and middle-income countries, to strengthen their health workforce and health systems.
